# Continuous low-dose cyclophosphamide plus prednisone in the treatment of relapsed and refractory multiple myeloma with severe complications

**DOI:** 10.3389/fonc.2023.1185991

**Published:** 2023-05-22

**Authors:** Haotian Shi, Wei Wei, Rong Peng, Haimin Chen, Nian Zhou, Lixia Wu, Wenjun Yu, Wenhao Zhao, Jian Hou, Fan Zhou

**Affiliations:** ^1^ Department of Hematologic Oncology, Zhabei Central Hospital in Shanghai Jing’an District, Shanghai, China; ^2^ Department of Hematology, Renji Hospital Affiliated to the School of Medicine, Shanghai Jiaotong University, Shanghai, China

**Keywords:** cyclophosphamide, prednisone, RRMM, response to therapy, survival, safety

## Abstract

**Background/objective:**

We retrospectively analyzed the effective and safety of continuous low-dose cyclophosphamide combined with prednisone (CP) in relapsed and refractory multiple myeloma (RRMM) patients with severe complications.

**Methods:**

A total of 130 RRMM patients with severe complications were enrolled in this study, among which 41 patients were further given bortezomib, lenalidomide, thalidomide or ixazomib on the basis of CP regimen (CP+X group). The response to therapy, adverse events (AEs), overall survival (OS) and progression-free survival (PFS) were recorded.

**Results:**

Among the 130 patients, 128 patients received therapeutic response assessment, with a complete remission rate (CRR) and objective response rate (ORR) of 4.7% and 58.6%, respectively. The median OS and PFS time were (38.0 ± 3.6) and (22.9±5.2) months, respectively. The most common AEs were hyperglycemia (7.7%), pneumonia (6.2%) and Cushing’s syndrome (5.4%). In addition, we found the pro-BNP/BNP level was obviously decreased while the LVEF (left ventricular ejection fraction) was increased in RRMM patients following CP treatment as compared with those before treatment. Furthermore, CP+X regimen further improved the CRR compared with that before receiving the CP+X regimen (24.4% *vs*. 2.4%, *P*=0.007). Also, both the OS and PFS rates were significantly elevated in patients received CP+X regimen following CP regimen as compared with the patients received CP regimen only.

**Conclusion:**

This study demonstrates the metronomic chemotherapy regimen of CP is effective to RRMM patients with severe complications.

## Introduction

Multiple myeloma (MM) is a clonal plasma cell neoplasm that accounts for about 1% of the malignancies and 10% of the hematologic malignancies worldwide, which often affects the elderly with an increased incidence year by year (1, 2). Owing to the advent of bortezomib, ixazomib, lenalidomide and other targeted drugs, the overall survival (OS) for MM patients has been markedly improved (3–5). However, MM still remains not cured and almost all of the patients will develop relapse/refractory MM (RRMM) with poor prognosis (6, 7). Thus, it is important to search effective treatment regimens to improve the prognosis of RRMM with severe complications.

In 2000, Harahan et al. (8) first proposed the concept of metronomic chemotherapy, referring continuous low-dose chemotherapy (LDM), with the advantages of low toxic and side effects. In addition, LDM can be used for long time due to the low dosage and shows higher antiangiogenic activity (9), which has been applied for the treatment of MM (10–12). It has been reported that combination of continuous low-dose cyclophosphamide and prednisone (CP) demonstrates significant anti-tumor activity in RRMM (13–15). For example, de Weerdt et al. (13) explored the effective and safety of continuous low-dose CP in 42 MM patients with progressive disease after melphalan-based and VAD (vincristine, adriamycin, and dexamethasone) treatment. The objective response rate (ORR) was 69% (29/42), and the median OS was 22.2 and 3.5 months for the responders and non-responders, respectively, with limited side-effects.

In this study, we clarified the effective and safety of continuous low-dose CP in the treatment of RRMM with severe complications in larger samples, as well as explored the prognostic influencing factors.

## Patients and methods

### Patients

Patients with RRMM and serious complications admitted to our hospital from January 2005 to October 2021 were included in this study. The inclusion criteria were (1): Patients met the diagnostic criteria of RRMM according to the International Myeloma Working Group (16) (2); Age >18 years old (3); Patients were ineligible to receive traditional chemotherapy due to at least one of the following conditions, heart failure NYHA (New York Heart Association) III ~ IV, lung function impairment with a grade of III ~ IV (17), coronary heart disease (CHD), hypertensive heart disease (HHD), hydropericardium, pleural effusion, seroperitoneum, Eastern Cooperative Oncology Group (ECOG) performance score ≥ 3 points or Karnofsky (KPS) score ≤ 40 points (4); Patients received at least one line (1–4) regimen for 1 to 8 courses before receiving low-dose CP regimen. Physical performance was assessed based on the ECOG standards (18). CD138 immunomagnetic beads were used to separate myeloma cells after 2015. MM risk stratification was performed according to Mayo Stratification of Myeloma and Risk-Adapted Therapy (mSMART) consensus guidelines 2013 (19). In detail, the patients were divided into high risk, intermediate risk and standard risk groups.

### Treatment methods

All patients were given continuous oral administration of low-dose CP (cyclophosphamide, 50 mg/d; prednisone,15 mg/d). Novel drugs including bortezomib, lenalidomide, thalidomide or ixazomib was given on the basis of CP (CP+X group) if the patients achieved improvements in organ failure (NYHA grade or lung function grade improved ≥ 1) or physical performance (ECOG score improvement ≥1 point or KPS score improvement ≥20 points) following CP regimen. Consideration of the general condition of the patient being poor, we reduced the therapeutic dose of novel drugs, specifically, bortezomib was given at a dose of 1.3 mg/m^2^ through intravenous infusion every week for all time, lenalidomide was given at a dose of 10 mg orally on day1-21, thalidomide was given at a dose of 100 mg orally every day for all time, and Isazzomib was given 3 mg orally 3 times every month (1 time/week). Patients were continued to receive CP regimen if the drugs were effective and no obvious adverse reaction was occurred, while the CP regimen was discontinued when the number of neutrophils and platelets decreased to 0.5×10^9^/L and 30×10^9^/L, respectively, or the patients failed to achieve disease stability (SD) after 3 months of standard therapy. Meanwhile, the patients were administered with granulocyte colony-stimulating factor (G-CSF) and apheresis platelets. The patients continued to receive CP regimen when neutrophils and platelets restored at the levels of >1.0×10^9^/L and 30×10^9^/L, respectively.

### Response to therapy and adverse events

The treatment response was assessed according to the International Myeloma Working Group consensus criteria for response (20), including complete response (CR), very good partial response (VGPR), partial response (PR), SD and progressive disease (PD). The changes of B-type natriuretic peptide (BNP)/pro-BNP and left ventricular ejection fraction (LVEF) in patients with cardiac insufficiency were assessed. AEs were evaluated according to the National Cancer Institute Standard for Common Toxic Events (NCICTCAE) 3.0. The ORR refers to the proportion of patients with sustained tumor shrinkage, including patients achieved CR and PR, in detail, ORR (%) = (CR+VGPR+PR)/(CR+VGPR+PR+SD+PD)×100%.

### Statistical analysis

SPSS 21.0 software was used for statistical analysis. Wilconxon matched test was used to compare the BNP/pro-BNP levels of patients before and after treatment. Differences of the clinical features and the incidences of AEs in the CP and CP+X groups were compared using the χ^2^ tests or Fisher’s exact tests. Kaplan-Meier curves with log-rank tests were used to analysis the influencing factors of progression-free survival (PFS) and OS. Univariate Cox was used to assess the prognostic influencing factors. *P*<0.05 was considered as statistical significance. PFS was defined as the time from diagnosis to the occurrence of PD, recurrence, death or the end of follow-up. OS was defined as the time from diagnosis to death from any cause or the end of follow-up.

## Results

### Patient clinicopathological characteristics

A total of 130 RRMM patients with seven complications were enrolled in this study, among which 89 patients received CP regimen and 41 patients received CP+X regimen following CP treatment. Among the 130 patients, 73 patients (56.6%) were male, 92 patients (71.3%) aged >65 years, and the immunoglobulin type of 20 patients (15.4%) were light chain type. In addition, most of the patients (95.3%; 122/128) were diagnosed at a stage of DS (Durie-Salmon) III, and 98 patients (76.6%) were diagnosed at a stage of III according to the ISS (International Staging System). Among the 130 patients, 72 patients had available FISH results, with 60 patients (83.3%) of standard-risk group, 5 patients (6.9%) in the intermediate-risk group and 7 patients (9.7%) in the high-risk group according to the mSMART 2013. The detailed clinical information of patients was demonstrated in **Supplementary Table 1**.

Among the 130 patients with RRMM, 41 patients received CP+X regimen because they achieved improvements in organ failure or physical performance following CP treatment. The gender, age, immunoglobulin type, DS stage, ISS stage, mSMART, and B2MG (β2-microglobulin) level at the time of CP treatment showed no obvious difference between the 2 groups, while the proportion of patients with NYHA I-II grade in the CP+X group was higher than that of the CP group (57.9% *vs*. 19.8%, *P*<0.001) (**Table 1**). This result indicated that NYHA grade maybe a factor of organ failure or physical performance improvement following CP regimen in RRMM patients.

**Table 1:** The clinicopathological characteristics of 130 patients with RRMM, n (%).

Characteristics	CP (n=89)	CP+X (n=41)	*P*
Gender			0.124
Male	46 (51.7)	27 (67.5)	
Female	43 (48.3)	13 (32.5)	
unknown	0	1	
Age			0.832
≤65	25 (28.1)	12 (30.0)	
>65	64 (71.9)	28 (70.0)	
unknown	0	1	
Heavy chain type			0.157
IgA	23 (25.8)	9 (22.0)	
IgD	8 (9.0)	2 (4.9)	
IgG	47 (52.8)	20 (48.8)	
IgM	1 (1.1)	0 (0.0)	
Undetectable	10 (11.2)	10 (24.4)	
Light chain type			1.000
κ	42 (47.2)	19 (47.5)	
λ	47 (52.8)	21 (52.5)	
unknown	0	1	
DS stage			0.666
I-II	5 (5.6)	1 (2.6)	
III	84 (94.4)	38 (97.4)	
unknown	0	2	
ISS			0.374
I-II	23 (25.8)	7 (17.9)	
III	66 (74.1)	32 (82.1)	
unknown	0	2	
NYHA			<0.001
I-II	17 (19.8)	22 (57.9)	
III-IV	69 (80.2)	16 (42.1)	
unknown	3	3	
B2MG			0.356
<3.5 μg/L	33 (41.3)	12 (33.3)	
3.5~5.5 μg/L	25 (31.3)	9 (25.0)	
>5.5 μg/L	22 (27.5)	15 (41.7)	
unknown	9	4	
Treatment methods before enrollment			0.173
1-2	59 (71.1)	31 (83.8)	
3-4	24 (28.9)	6 (16.2)	
unknown	6	4	
mSMART			0.890
Standard-risk	27 (87.1)	33 (80.5)	
Intermediate-risk	2 (6.5)	3 (7.3)	
High-risk	2 (6.5)	5 (12.2)	
unknown	58	0	

B2MG, β2-microglobulin; DS, Durie-Salmon; ISS, International Staging System.

### Effectiveness

**Figure 1:**
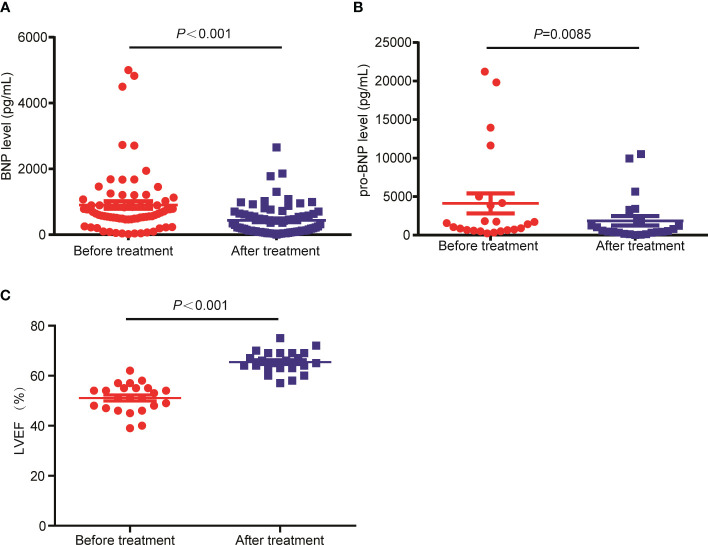
The levels of BNP and pro-BNP were decreased in RRMM patients following CP treatment. **(A)** The BNP, **(B)** pro-BNP and **(C)** LEVF levels were tested before and after CP treatment.

The therapeutic response of 128 of the 130 patients were evaluated as 2 patients were lost to follow up at the time of efficacy assessment, among which 6 patients (4.7%) achieved CR, 19 patients (14.8%) achieved VGPR, 50 patients (39.1%) achieved PR, 19 patients (14.8%) achieved SD, with the remaining 34 patients (26.6%) achieved PD following CP regimen. The CRR and ORR were 4.7% and 58.6%, respectively. The drug onset time which refers to the time from the start of using CP regimen to the time of disease being controlled was 1-4 months, with a median drug onset time of 2 months. In addition, we assessed the effect of CP treatment on the heart function of patients with RRMM. The BNP level, a marker of heart function (21), was detected in 73 patients before CP treatment and after CP treatment. Also, we tested the level of pro-BNP in 23 patients before and after CP regimen. Compared with those before CP treatment, the levels of pro-BNP (**Figure 1A**) and BNP (**Figure 1B**) were significantly decreased after CP treatment. In addition, the LVEF was significantly increased following the treatment (**Figure 1C**). These results suggested that CP treatment could improve the heart function of MM patients.

**Figure 2:**
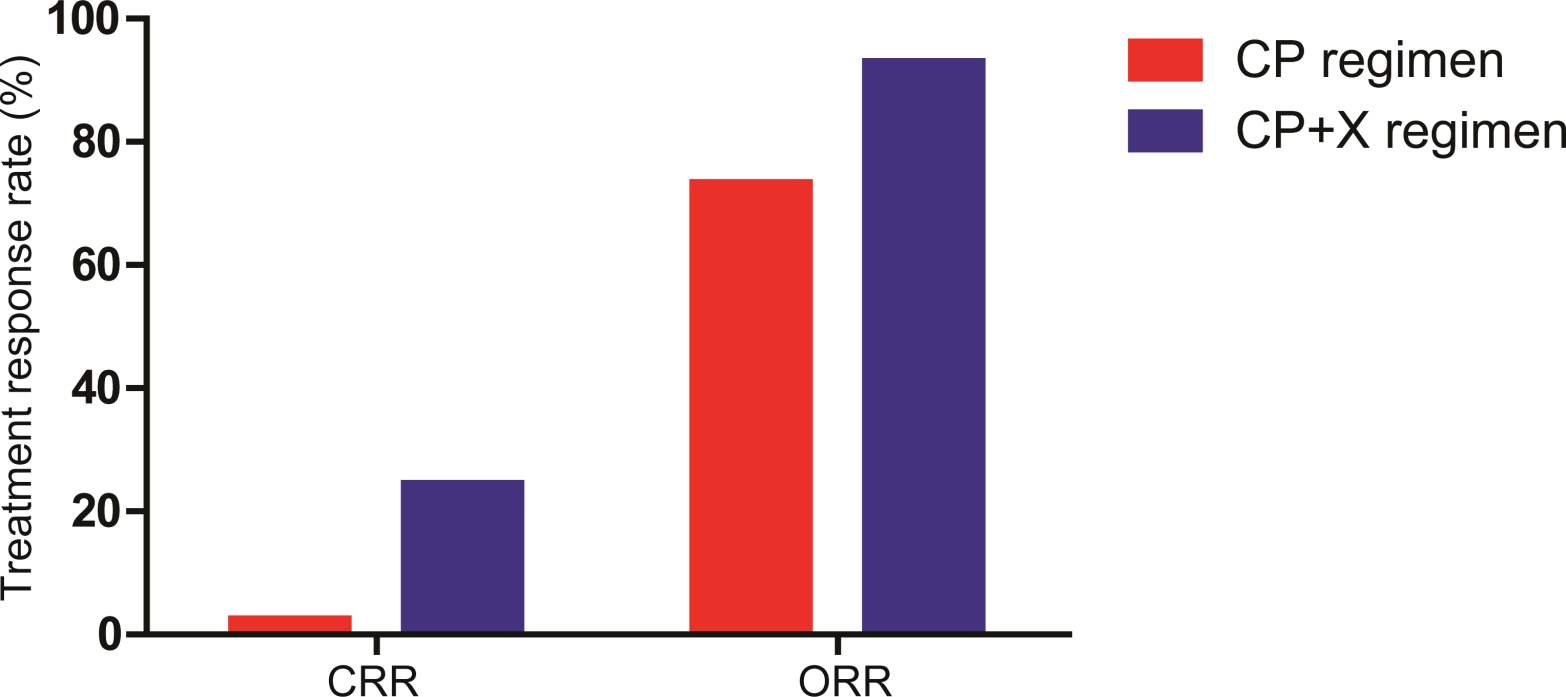
The CRR and ORR of patients received CP and CP+X. Both CRR and ORR were significantly increased following CP+X treatment.

All 41 patients in the CP+X group were evaluated for therapeutic response to CP and CP+X regimens. Among them, 1 patient (2.4%) achieved CR, 11 patients (26.9%) achieved VGPR, 18 patients (43.9%) achieved PR, 8 patients (19.5%) achieved SD, and 3 patients (7.3%) achieved PD following CP regimen. The CRR and ORR were 2.4% and 73.2%, respectively. Following CP+X regimen, 10 patients (24.4%) achieved CR, 14 patients (34.1%) achieved VGPR, 10 patients (24.4%) achieved PR, 4 (9.8%) and 3 patients (7.3%) achieved SD and PD, respectively. The CRR and ORR rate of CP+X were 24.4% and 92.9%, respectively. The CRR was significantly increased following CP+X regimen as compared with that before CP+X regimen (24.4% *vs*. 2.4%, *P*=0.007) (**Figure 2**).

### Safety

During the treatment period, 41 (31.5%) of the 130 patients occurred AEs, including 10 cases (7.7%) of hyperglycemia, 8 cases (6.2%) of pneumonia, 7 cases (5.4%) of Cushing’s syndrome, 6 cases (4.6%) of gastritis, 4 cases (3.1%) of infection, 1 case (0.8%) of hypokalemia, 1 case (0.8%) of osteonecrosis of the femoral head (ONFH), 1 case (0.8%) of paronychitis combined with osteomyelitis, 1 case (0.8%) of limb numbness, 1 case (0.8%) of hemoptysis, and 1 case (0.8%) of gastrointestinal bleeding.

In addition, we compared the incidence of AEs between CP and CP+X groups during the treatment period. AEs were found in 20 patients (48.8%) of the CP+X group, which was significantly higher than that of the CP group (23.6%; 21/89) (*P*=0.004), especially for hyperglycemia (17.1% *vs*. 3.4%, *P*=0.011), **Table 2**.

**Table 2:** AEs of the CP and CP+X groups, n (%).

AEs	CP (n=89)	CP+X (n=41)	*P*
Hyperglycemia	3 (3.4)	7 (17.1)	0.011
Pneumonia	7 (7.9)	1 (2.4)	0.217
Cushing’s syndrome	3 (3.4)	4 (9.8)	0.206
Gastritis	2 (2.2)	4 (9.8)	0.078
Infection	4 (4.5)	0 (0.0)	0.307
Hypokalemia	0 (0.0)	1 (2.4)	0.315
Osteonecrosis of the femoral head	0 (0.0)	1 (2.4)	0.315
Paronychitis combined with osteomyelitis	0 (0.0)	1 (2.4)	0.315
Limb numbness	1 (1.1)	0 (0.0)	1.000
Hemoptysis	1 (1.1)	0 (0.0)	1.000
Gastrointestinal bleeding	0 (0.0)	1 (2.4)	0.315
Total	21 (23.6)	20 (48.8)	0.004

### Survival

The median follow-up time for all of the 130 patients was (110.0 ± 6.6) months, with a 9-year OS and PFS rates of (12.4 ± 3.4)% and (21.3±3.8), respectively. The time to last full examination was 17 July 2022, and 2 of the 130 patients (1 of the CP group and 1 of the CP+X group) were lost to follow up during the study. The median OS and PFS time were (38.0 ± 3.6) months and (22.9±5.2) months, respectively.

**Figure 3:**
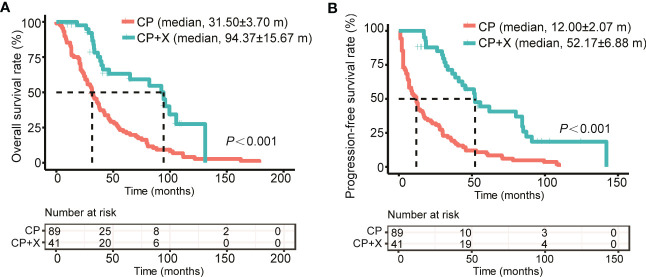
Comparison of the survival rates of RRMM patients treated with CP and CP+X. Kaplan-Meier curves were used to assess the **(A)** OS and **(B)** PFS of RRMM patients treated with CP or CP+X. (m, months).

Also, we compared the survival of patients received CP only (CP group) and CP+X following CP regimen (CP+X group). The median follow-up time for patients in the CP+X group was (93.87 ± 19.68) months, with a 9-year survival rate of (27.5 ± 10.1)%, and the median survival time of (94.37 ± 15.67) months, respectively. For the CP group, the 9-year survival rate was (6.8 ± 2.8)%, and the median survival time was (31.5 ± 3.7) months. Both the OS and PFS rates of the CP+X group significantly higher than those of the CP group (**Figures 3A, B**).

### Prognosis analysis

Moreover, we analyzed the prognostic influencing factors of RRMM using the univariate Cox analysis. Among the factors of gender, age, globulin type, DS, ISS and B2MG level, number of complications (4–7), heart failure, lung function (III-IV), CHD, HHD, arrhythmia, diabetes, hydropericardium, lung infection and pleural effusion at the time of CP treatment, the light chain type of globulin was a protective factor (*P*=0.037) while heart failure was a worse factor of PFS (*P*=0.024) for patients with RRMM (**Table 3**).

**Table 3:** Univariate Cox analysis of the prognosis influencing factors of patients with RRMM.

Factors	PFS			OS			
	HR	95%CI	*P*	HR	95%CI	*P*	
Gender (male)	0.989	0.679, 1.440	0.953	1.342	0.905, 1.990	0.143	
Age (>65 years)	1.00	0.668, 1.519	0.972	1.068	0.697, 1.636	0.762	
Globulin (Light chain type)	0.538	0.300, 0.962	0.037	0.723	0.409, 1.278	0.265	
DS (III-IV)	1.752	0.830, 3.698	0.142	1.903	0.758, 4.780	0.171	
ISS (III-IV)	1.280	0.828, 1.977	0.267	1.166	0.749, 1.816	0.497	
B2MG (elevated)	0.862	0.536, 1.387	0.541	1.343	0.801, 2.251	0.263	
Number of complications (4–7)	0.837	0.484, 1.446	0.524	1.166	0.640, 2.125	0.616
Heart failure	1.705	1.073, 2.711	0.024	1.225	0.756, 1.985	0.410
Lung function (III-IV)	1.055	0.709, 1.569	0.791	1.342	0.883, 2.038	0.168
CHD	1.138	0.781, 1.659	0.500	0.994	0.672, 1.470	0.975
Hypertensive heart disease	0.831	0.562, 1.230	0.355	1.243	0.551, 1.177	0.362
Arrhythmia	0.938	0.631, 1.395	0.752	0.794	0.702, 1.590	0.794
Diabetes	0.672	0.430, 1.049	0.080	0.465	0.534, 1.332	0.843
Hydropericardium	0.773	0.338, 1.769	0.543	0.359	0.294, 1.557	0.677
Lung infection	1.157	0.548, 2.443	0.702	2.065	0.820, 5.198	0.124
Pleural effusion	0.858	0.510, 1.444	0.565	0.997	0.585, 1.688	0.991

B2MG, β2-microglobulin; DS, Durie-Salmon; ISS, International Staging System; CHD, Coronary heart disease.

## Discussion

Daily oral administration of low-doses of CP is a form of metronomic chemotherapy, which shows good efficacy against refractory B-cell malignancies though inhibiting endothelial cell activation and anti-tumor angiogenesis (22, 23). Fortunately, combination of low-dose CP with other low toxicity targeted drugs such as bortezomib, lenalidomide or pomalidomide, can achieve a higher ORR of 65-73% (24–26). Herein, we retrospectively analyzed the effective and safety of continuous low-dose CP in the treatment of RRMM with severe complications in a larger cohort.

Previously, our group demonstrated that low-dose CP regimen showed a good efficacy in the treatment of RRMM with severe heart failure in a small cohort study (n=56) with the CRR and ORR of 3.7% and 59.2% (15). To further verify the effectiveness of CP metronomic chemotherapy, we further assessed its efficacy in larger size of RRMM patients with severe complications, including but not limited to heart failure, with other complications of lung function impairment, CHD, HHD, arrhythmia, diabetes, hydropericardium, pulmonary infection and hydrothorax. The CRR and ORR were 4.7% and 58.6%, which were close to our earlier report (15). Consistently (15), we observed that the heart function of RRMM patients was obviously improved following CP regimen with decreased BNP/pro-BNP and increased LVEF levels. Severe complications significantly limit the treatment options of RRMM patients, and the metronomic chemotherapy application provide a treatment choice. For example, Dimopoulos et al. (27) prospectively evaluated the efficacy of pomalidomide plus low-dose dexamethasone in RRMM patients with moderate or severe renal injury (RI). The ORRs were 39.4%, 32.4%, and 14.3% for patients with moderate RI, severe RI and severe RI, with the median OS of 16.4 months, 11.8 months, and 5.2 months, respectively. Herein, we showed that the median OS and PFS of RRMM patients with severe complications were (38.0 ± 3.6) months and (22.9±5.2) months following the treatment of low-dose CP regimen.

The advent of novel drugs such as lenalidomide and bortezomib has dramatically improved the survival of patients with RRMM. Recently, Fazio et al. (28) explored the effectiveness of daratumumab combined with dexamethasone and lenalidomide or bortezomib in RRMM patients, and the CRR and ORR were 11% and 84%, respectively. In this study, patients achieved improvements in organ failure or physical performance following CP regimen were given bortezomib, lenalidomide/thalidomide or ixazomib based on CP regimen. Both the CRR and ORR were improved following CP+X treatment, as well as the OS and PFS. Noticeably, 6 (54.5%) of 11 patients received lenalidomide/thalidomide achieved CR. Thalidomide and lenalidomide are the first- and second-generation immunomodulatory drugs (IMiD), which are widely used in the treatment RRMM and significantly improve the prognosis of patients (29, 30). These findings provide solid evidence for the use of low-dose CP combined with novel drugs in RRMM patients with severe complications.

Two main limitations must be clarified of the manuscript. One is the sample size although it is the study with largest cohort, and we intend to verify the efficacy of CP regimen in larger populations. In addition, the patients who achieved improvements in organ failure or physical performance following CP regimen were given CP+X regimen, thus the efficacy of “CP+X” on RRMM remains unclear. It is worthy to assess the efficacy and safety of “CP+X” on RRMM patients, aiming to provide potent treatment strategy for RRMM patients.

## Conclusion

CP metronomic chemotherapy is an effective treatment method for RRMM patients with severe complications. Combination of new drugs such as bortezomib, lenalidomide, thalidomide or ixazomib with CP can achieve better efficacy if the patients’ economic and physical conditions permit.

## Data availability statement

The original contributions presented in the study are included in the article/**Supplementary Material**. Further inquiries can be directed to the corresponding authors.

## Ethics statement

The studies involving human participants were reviewed and approved by Zhabei Central Hospital in Shanghai Jing’an District. The patients/participants provided their written informed consent to participate in this study.

## Author contributions

HS, JH, and FZ conceived and designed the study. All authors provided study material or patients, collected or assembled data, participated in drafting and revising the manuscript, approved the final version of the manuscript, and are accountable for all aspects of this work.

## Supplementary material

Supplementary Table 1 Clinicopathological information of the 130 patients with RRMM.
